# Alzheimer’s disease: risk factors and potentially protective measures

**DOI:** 10.1186/s12929-019-0524-y

**Published:** 2019-05-09

**Authors:** Marcos Vinícius Ferreira Silva, Cristina de Mello Gomide Loures, Luan Carlos Vieira Alves, Leonardo Cruz de Souza, Karina Braga Gomes Borges, Maria das Graças Carvalho

**Affiliations:** 1 Faculdade de Farmácia, Universidade Federal de Minas Gerais, Avenida Presidente Antônio Carlos, 6627 – Pampulha, Belo Horizonte, Minas Gerais 31270-901 Brazil; 2 Faculdade de Medicina, Universidade Federal de Minas Gerais, Av. Prof. Alfredo Balena, 190 - Santa Efigênia, Belo Horizonte, Minas Gerais 30130-100 Brazil

**Keywords:** Alzheimer’s disease, Genetic risk factors, Acquired risk factors

## Abstract

Alzheimer’s disease (AD) is the most common type of dementia and typically manifests through a progressive loss of episodic memory and cognitive function, subsequently causing language and visuospatial skills deficiencies, which are often accompanied by behavioral disorders such as apathy, aggressiveness and depression. The presence of extracellular plaques of insoluble β-amyloid peptide (Aβ) and neurofibrillary tangles (NFT) containing hyperphosphorylated tau protein (P-tau) in the neuronal cytoplasm is a remarkable pathophysiological cause in patients’ brains. Approximately 70% of the risk of developing AD can be attributed to genetics. However, acquired factors such as cerebrovascular diseases, diabetes, hypertension, obesity and dyslipidemia increase the risk of AD development. The aim of the present minireview was to summarize the pathophysiological mechanism and the main risk factors for AD. As a complement, some protective factors associated with a lower risk of disease incidence, such as cognitive reserve, physical activity and diet will also be addressed.

## Introduction

Alzheimer’s disease (AD) is the most common type of dementia [1], affecting at least 27 million people and corresponding from 60 to 70% of all dementias cases [2]. The occurrence of this disease also has a huge impact on life of patient’s family, in addition to a high financial cost to society [3]. From an anatomopathological point of view, AD is characterized by two prototypical lesions: 1) senile plaques, composed of a nucleus of β-amyloid protein accumulation (Aβ42), as extra-cellular lesions and 2) neurofibrillary tangles composed of phosphorylated tau protein (P-tau) and which are intraneuronal findings [4]. Deposition of β-amyloid protein can also occur in capillaries walls, arteries and arterioles causing amyloid cerebral angiopathy leading to degeneration of vascular wall componentes and worsening of blood flow, besides predisposing to intraparenchymal hemorrhages [5].

AD typically manifests through a progressive loss of episodic memory and cognitive function, with later deficiency of language and visuospatial abilities. Such changes are often accompanied by behavioral disorders such as apathy, aggressiveness and depression [6]. It should be noted that there is an important subgroup of AD patients who do not present a typically amnestic picture, manifesting non-amnestic deficits from the onset of symptoms [7]. Structural neuroimaging, with a pattern of hippocampal and parietal atrophy in typical cases reinforces the diagnosis [8]. Patients who meet typical disease characteristics, excluding other causes such as vascular and fronto-temporal dementias, have a probable diagnosis of AD [6]. Definitive diagnosis of the disease is usually carried out only through postmortem examination, whose purpose is to demonstrate histologically the neurofibrillary tangles and the senile plaques [9].

## Pathophysiology of Alzheimer’s disease

The presence of extracellular plaques of insoluble β-amyloid peptide (Aβ) and neurofibrillary tangles (NFT) of P-tau in neuronal cytoplasm is the hallmark of AD [10]. Although the mechanisms by which these changes lead to cognitive decline are still debated, these deposits are believed to lead to atrophy and death of neurons resulting from excitotoxicity processes [excessive stimulation of neurotransmitter receptors in neuronal membranes], collapse in calcium homeostasis, inflammation and depletion of energy and neuronal factors. As a result of this process, damage to neurons and synapses involved in memory processes, learning and other cognitive functions lead to the aforementioned cognitive decline [11].

According to amyloid cascade theory (one of the most accepted theories about AD pathogenesis, although still debated), the cerebral accumulation of Aβ peptide, resulting from the imbalance between production and clearance of this protein, is the main event causing the disease, being other events observed (including the formation of NFT) resulting from this process [12].

The Aβ peptide, which has 36 to 43 aminoacids, is derived from amyloid precursor protein (APP) enzymatic proteolysis, a physiologically produced protein that plays important roles in brain homeostasis [13, 14]. The *APP* gene is located on chromosome 21, which explains the higher incidence of early-onset AD in individuals with 21 trisomy (Down Syndrome) and in individuals with *APP* gene locus duplication [a rare form of early onset of familial origin]. It is believed that overexpression of APP results in an increase of cerebral Aβ peptide, and consequently, in its deposition [15].

Two main pathways for APP processing are now recognized: a non-amyloidogenic α-secretase-mediated pathway and an amyloidogenic β-and γ-secretase-mediated pathway. Cleavage of APP by α-secretase results in a soluble molecule, sAPPα, which has probable neuroprotective function, playing important roles in the plasticity and survival of neurons and protection against excitotoxicity [16, 17]. The Aβ peptide is produced by APP cleavage by a β-secretase (mainly BACE1 enzyme). In this pathway, APP is cleaved by β-secretase to give a APP soluble fragment (sAPPβ, a mediator related to neuronal death), and a carboxy-terminal complex linked to cell membrane. The latter is cleaved by a γ-secretase complex composed by 4 proteins: presenilin 1 or 2, nicastrin, APH-1 (formerly pharynx-defective-1) and and PEN-2 (presenilin enhancer-2), to give rise to the Aβ peptide. Aβ peptides ranging in size from 38 to 43 aminoacids are generated with predominance of the 40 aminoacid form (Aβ 40), followed by 42 (Aβ 42) [17, 18]. In physiological conditions, the amyloidogenic and non-amyloidogenic pathways coexist in equilibrium, the latter being favored preferentially [19].

The Aβ42 peptide is more prone to aggregation than Aβ40. Immunohistochemical analyses indicate that Aβ42 is initially deposited and found at higher concentrations in the amyloid plaques observed in AD patients [20]. Several studies showed that CSF Aβ42 levels are surrogate markers of underlying brain amyloidosis [21, 22]. On the contrary, the correlation between serum Aβ42 levels and cerebral amyloidosis is not yet demonstrated. A decrease in Aβ42 levels is observed in cerebrospinal fluid of AD subjects, which can be explained in part by higher deposition of β-amyloid plaques [23]. As additional evidence of Aβ42 peptide and the AD pathophysiology, it is further noted that mutations in APP and presenilin genes, which give rise to early-onset familial AD forms, lead to a relative increase in Aβ42 levels [20].

Aβ peptides, under physiological conditions, are produced primarily in monomeric forms with synapses protective function. However, the accumulation of this protein leads to formation of fibrils that accumulate in senile plaques. High levels of Aβ may lead to oligomeric products formation (dimers, trimers, tetramers) leading to neuronal toxicity and degeneration (both by interaction with cell membranes and their receptors, and by direct interference in intracellular processes), interfering with the function and survival of cholinergic, serotonergic, noradrenergic and dopaminergic neurons, reducing their control over the amyloidogenic pathway and favoring the accumulation of insoluble Aβ peptide [19, 24].

The exact mechanism by which deposition of Aβ peptide promotes NFT formation of hyperphosphorylated tau protein is not known. Blurton-Jones & Laferla (2006) [25] suggest four basic mechanisms:

- The Aβ peptide promotes the activation of specific kinases (GSK3β, e.g.) that catalyze the hyperphosphorylation of tau protein, leading to its conformation change and formation of NFT;
- Neuroinflammation promoted by the deposition of Aβ peptide leads to the production of proinflammatory cytokines that stimulate the phosphorylation of tau protein;
- Reduced capacity of degradation of tau protein by the proteasome, in a process induced by Aβ peptide;
- Defects in axonal transport promoted by Aβ peptide lead to inadequate localization of tau protein and its messenger RNA, which can lead to hyperphosphorylation and aggregation in NFT.

Tau protein is a microtubule-associated protein, produced by alternative splicing of the *MAPT* gene, located on chromosome 17 (17q21). Six isoforms of tau protein are produced by this process [26]. The main known physiological functions of this protein are the stimulation of tubulin polymerization, microtubules stabilization and intracellular organelles transport by microtubules. Once hyperphosphorylated, the protein loses its functions in the synthesis and stabilization of microtubules, leading to neuronal damage and promoting cytotoxicity [27]. Histological analyses demonstrate that both the load and the distribution of NFT in brain tissue correlate better with the severity of cognitive deficit than the Aβ peptide deposits [28].

## Genetic risk factors

AD can be classified by the age of onset of the first symptoms. Early-onset AD affects individuals under 65 years of age, accounting for about 4–6% of cases of AD, while the late form AD affects individuals aged 65 years or older. Besides the age of onset of symptoms, the early and late forms of AD differ in other clinical, neuropsychological, neuropathological and neuroimaging variables [29].

According to Ballard et al. (2011) [1] about 70% of the risk of developing AD can be attributed to genetics. Early AD usually occurs due to mutations in genes *APP, PSEN1* and *PSEN2* (genes of amyloid precursor protein, presenilin 1 and presenilin 2, respectively), whereas late-form AD is mainly associated with a polymorphism in *APOE* gene (apolipoprotein E gene), especially the presence of ε4 allele [30, 31].

More than 30 dominant mutations have already been found in *APP* gene (located in chromosome 21q21) and are associated with about 15% of cases of early-onset autosomal dominant AD. Mutations in *PSEN1* gene (located at 14q24.3) are associated with 80% of cases of early-onset AD, whereas 5% of cases are associated with *PSEN2* mutations (located at 1q31-q42) [32]. Most of *APP* gene mutations, as well as *PSEN1* mutations, lead to an increase in Aβ42: Aβ40 ratio, either by Aβ42 increased expression, reduction of Aβ40, or both. This deregulation favors early Aβ deposition in brain tissue favoring the amyloidogenic cascade [33]. It is believed that there are other genes besides APP, PSEN1 and PSEN2 involved in the pathogenesis of early-onset AD, as demonstrated by Campion et al. (1999) [34].

Apolipoprotein E (ApoE) is a protein involved in lipid metabolism encoded by *APOE* gene, located on chromosome 19. There are three *APOE* alleles described (ε2, ε3 and ε4, giving rise to apoE2, apoE3 and apoE4 isoforms), present in population at different frequencies (ε2: 5–10%, ε3: 65–70% and ε4: 15–20%). A study by Corbo and Scacchi (1999) [35] showed that there is a great variability in the APOE allele distribution among the different populations, with ε2 frequencies varying from 0.0 in some Native American populations up to 0.145 in Papuans. The ε 4 frequencies obtained by the authors range from 0.052 (Sardinians) to 0.407 (Pygmies). The ε4 allele is the main risk factor for late-onset AD. The presence of ε4 in heterozygosity increases 3-fold the risk of AD developing, whereas in homozygosis, the risk is increased 12-fold. Conversely, the presence of ε2 allele reduces the risk of AD developing [36, 37].

The causes of the association between apoE are not yet fully understood, although some mechanisms have been proposed, and presented consistent results in clinical and in vitro studies. Among these studies, some show that apoE is able to bind to Aβ peptide. While the apoE4 isoform binds to Aβ peptide promoting its polymerization in fibrils and its deposition, apoE2 and apoE3 forms are more efficient in promoting the clearance of this peptide, reducing its deposition in brain tissue [38]. ApoE has neuroprotective effects and is able to act on neurons development, with apoE2 and apoE3 performing better than apoE4. Additionally, it is observed that protease-generated apoE fragments have toxic effects, which may lead to neuronal injury and favor Aβ peptide deposition [38, 39].

More recently it was observed that rare alterations in the triggering receptor expressed on myeloid cells 2 (*TREM2*) gene elevated the risk ratio by 2.9% for AD development [40, 41]. The pathophysiological mechanism by which the deficiency in the gene increases the risk ratio for AD still needs to be better clarified. The gene is located on chromosome 6p21 [42] and the TREM2 protein is a highly expressed receptor on the surface of microglia, phagocytic cells of central nervous system, and has the function of modulating phagocytic and inflammatory responses in central nervous system [43]. Activation of microglia through the interaction of TREM2 and DAP12 stimulates the production of CCL19 and CCL21 chemokines and phagocytosis [44]. In knockout models for the TREM2 receptor it was observed that phagocytic capacity of apoptotic neuronal cell bodies was deficient [44]. Thus the accumulation of these cellular debris would promote a proinflammatory microenvironment [44]. Xiang et al. (2016) [45] observed that the removal capacity of Aβ peptide deposits is impaired in TREM2 receptor deficiency and would favor amyloid plaques accumulation.

## Acquired risk factors

A number of acquired factors increase the risk of developing AD. Among those factors are cerebrovascular diseases (most commonly reported risk factor), diabetes, hypertension, obesity and dyslipidemia [46]. The association of these risk factors to AD development will be described in the following subsections, as well as some protective factors associated with a lower risk of disease incidence, such as cognitive reserve, physical activity and diet as reported by Mayeux & Stern (2012) [46].

### Cerebrovascular diseases

Cerebrovascular diseases and AD share many risk factors, which often overlap. Cerebrovascular changes such as hemorrhagic infarcts, small and large ischemic cortical infarcts, vasculopathies, and changes in cerebral white matter are known to increase the risk of dementia. Postmortem analyses of the brains of patients with AD indicate the presence of parenchymal vascular disease (amyloid angiopathy by Aβ peptide and small vessels arteriolosclerotic disease), with hemorrhagic outbreaks and infarcts being found in more than 50% of them [47, 48]. According to Liu et al., (2015) [49], neuropathological findings indicate that between 6 and 47% of individuals with dementia have a simultaneous occurrence of cerebrovascular disease. These observations point to the potential role of homeostatic mechanisms in AD and lead to question whether the dementias in which vascular processes are involved are fundamentally different from those related to accumulation of Aβ42 and tau proteins or if both pathological processes produce synergistic effects on cognitive function [9].

According to the “double-stroke” theory of AD, vascular risk factors (“first stroke”) lead to dysfunction in blood-brain barrier and reduction in cerebral blood flow, with decreased blood supply to the region (oligoemia). This event leads to neuronal damage by non-amyloidogenic and amyloidogenic pathways. Firstly, the dysfunction of blood-brain barrier leads to oligoemia and the accumulation of neurotoxic molecules, events associated with the occurrence of multiple focal ischemic infarcts and micro-injuries resulting from hypoxia, causing neuronal damage. In the amyloidogenic pathway, vascular injury leads to increased expression and processing of APP, resulting in an increase in Aβ peptide. In addition, damage to blood-brain barrier leads to decreased clearance of Aβ peptide. The accumulation of amyloid in brain (“second stroke”) amplifies neuronal dysfunction and speeds up neurodegeneration process. Both Aβ peptide accumulation and hypoperfusion lead to hyperphosphorylation of tau protein, promoting the formation of NFT [50].

### Hypertension

A longitudinal study carried out by Skoog et al. (1996) demonstrated that hypertension is capable of leading to increased risk of developing AD [51]. Other studies have confirmed this association, indicating that hypertension, especially when present in middle age, negatively affects cognitive performance at more advanced ages, and this association becomes weaker with age [52]. Hypertension is capable of causing changes in the vascular walls which can lead to hypoperfusion, ischemia and cerebral hypoxia, contributing to trigger the development of AD. Studies demonstrate that cerebral ischemia is capable of leading to the accumulation of APP and Aβ, in addition to stimulating the expression of presenilin, involved in Aβ synthesis. Hypertension may also lead to dysfunction in the blood-brain barrier, an event associated with the genesis of AD by previously discussed mechanisms [53].

### Type 2 diabetes

Epidemiological studies indicate a clear association between type 2 diabetes mellitus and the increased risk of developing AD. Several mechanisms for this association are suggested, including insulin resistance and insulin deficiency, impaired insulin receptor, toxicity of hyperglycemia, adverse effects due to advanced glycation end products, cerebrovascular damage, vascular inflammation and others [54].

The use of animal models was able to demonstrate that deficiency or resistance to insulin are able to stimulate the action of β and γ-secretases, besides promoting reduction of Aβ clearance, leading to its accumulation in brain tissue. Insulin resistance or deficiency are still capable of inducing hyperphosphorylation of tau protein, leading to NFT formation. Insulin and insulin-like growth factor bind to insulin receptor, leading to its autophosphorylation and activation. Activation of this receptor leads to phosphorylation of phosphoinositide 3-kinase (PI3K) enzyme, which in turn phosphorylates and inhibits glycogen synthase kinase 3β (GSK3β) enzyme, which is important for tau protein phosphorylation. Thus, insulin deficiency / resistance leads to GSK3β abnormal activation, and consequently, to an increase of p-tau formation [55].

In addition to the mechanisms discussed earlier, studies have reported that advanced glycation end products (AGEs) induce neuronal death through activation of cell death pathways, in addition to stimulating APP processing through increased expression of complexes β and γ-secretases (BACE and PSEN1), in a process involving reactive oxygen species generation [56]. In addition, Aβ peptide may undergo non-enzymatic glycation, making it an AGE more neurotoxic than its non-glycated form [57].

### Obesity

The role of obesity as a risk factor for AD development is still uncertain, with studies presenting rather heterogeneous results. According to a meta-analysis developed by Profenno, Porsteinsson, & Faraone (2010) [58], obesity (Body Mass Index - BMI ≥30 kg / m^2^) is significantly and independently associated with AD developing risk. On the other hand, a meta-analysis conducted by Fitzpatrick et al. (2009) [59] indicated that obesity in middle age is a risk factor for dementia development (hazard ratio - HR: 1.39; 95% CI: 1.03–1.87), while in later stages of life, obesity is inversely correlated with the risk of dementia (HR: 0.63; 95% CI: 0.44–0.91). The same authors have also reported that below-ideal weight (BMI < 20 kg / m^2^) is also associated with an increased risk of dementia (HR: 1.62, 95% CI: 1.02–2.64). Weight loss at more advanced ages occurs in concomitance to other comorbidities and is often indicative of poor health, and may even precede dementia onset within 10 years. Another meta-analysis conducted by Anstey et al. (2011) [60] indicated that both low weight and overweight as well as obesity in middle age are associated with a higher risk of developing AD in late life.

### Dyslipidemia

Elevated cholesterol levels have been proposed as risk factors for the development of AD. Studies have already demonstrated 10% higher cholesterol levels in patients with AD, compared to healthy individuals [61]. Hypercholesterolemia is a risk factor both for atherosclerosis development and AD development as well as other neurodegenerative diseases [62].

Hypercholesterolemia increases AD risk primarily because of its effects on the blood-brain barrier. Studies have shown that elevated circulating cholesterol levels are capable of compromising integrity in blood-brain barrier [62], resulting in mechanisms previously discussed. In addition, experimental studies using animal models demonstrate that hypercholesterolemia is associated with increased Aβ peptide deposition, in addition to increased NFT formation, cognitive decline, neuroinflammation, dysfunction of cholinergic neurons and the presence of cerebral microhemorrhages compatible with AD [63, 64].

In observational studies a beneficial effect was observed in the users of statins as the reduction in AD incidence or improvement in the disease progression [65–67]. However, clinical studies to date have not demonstrated benefit of statins treatment and protection against cognitive decline in AD patients at various stages of disease [68–72]. Contrary to meta-analysis findings conducted by Song et al. (2013) [73] who observed a lower risk of developing AD in statins users, a Cochrane meta-analysis [74] did not observe difference in disease outcome as well as alteration in mini-mental status examination (MMSE) in patients using or not statins. However, some important questions regarding the clinical studies are pointed out, i.e., whether treatment initiated in middle age prior disease onset would also have a beneficial effect in elderly, or whether in people with AD family history the treatment would be effective in comparison to those without this background.

### Marital status, stress, depression and inadequate sleep

Widowhood status has been reported as an important risk factor AD. A cohort study by Håkansson et al. (2009) [75] shows that widowed individuals have an increased risk of developing AD compared to married or cohabiting individuals and that this effect is more pronounced in carriers of the APOE ε4 allele. Other studies, such as that by Fan et al. (2015) [76] demonstrated an association between the risk of all-cause dementia and widow status. A meta-analysis by Sommerlad et al. (2018) [77] reported an association between widowhood and all-cause dementia, but the same association was not found between widowhood and AD or vascular dementia.

Studies in animal models of AD have shown that stress, characterized as hyperactivation of the hypothalamic, pituitary and adrenal axis (HPA) leading to an increase in cortisol production, causes an increase in Aβ peptide deposition in regions of the brain such as hypothalamus and prefrontal cortex [78–80]. Carroll et al. (2011) [81] have observed that the prolonged stress caused by this hyperactivation also causes an increase in the accumulation of hyperphosphorylated tau and neurodegeneration in mice. In humans, increased levels of cortisol were observed in patients with AD compared to the control group [82–84]. Huang et al. (2009) [85] observed in a 2-year follow-up of patients with AD that the higher cortisol levels correlated with the faster progression of the disease, worsened in the MMSE and smaller volume of the hippocampus region when observed by resonance. The authors of this study argue that hippocampal atrophy causes a disinhibition effect on the HPA axis, which would cause elevation in cortisol levels as a consequence of the pathophysiological process of AD. Toledo et al. (2012) [86], observed in a sample of 26 patients with AD that the increase in cortisol levels is correlated with the deposition of the Aβ peptide observed by means of pittsburgh compound b-positron emission tomography (PiB-PET). Ennis et al. (2017) [87], in a 10-year longitudinal study with 1025 participants observed an increased risk of 1.31 for the development of AD and elevation in cortisol levels that were dosed in 24-h urine samples. However, this result contrasts with that observed in the Rotterdan study [88] in blood samples collected in the morning when there was no correlation between cortisol levels and AD or dementia in general.

Early adult depression is a risk factor for the development of dementia at more advanced age including AD [89–91]. Zverova et al. (2013) [83] observed a greater odds ratio for cognitive decline in the presence of cortisol levels and patients with AD and symptoms of depression. Wu et al. (2018) [92] observed in some patients with major depression in middle age hippocampal atrophy and Aβ peptide deposition observed by PET indicating that the protein metabolism may be altered in patients with depression.

According to a study published by Proserpio et al. (2018) [93], sleep disorders have a bidirectional relationship with AD: sleep disorders arise during the early stages of dementia and tend to worsen with the onset of dementia. Similarly, sleep disorders can lead to an increased risk of dementia. A meta-analysis by Shi et al. (2018) [94] demonstrated that individuals with sleep disorders have an increased risk of developing dementia. More specifically, individuals with insomnia are at high risk for developing AD but not for vascular dementia or other causes. Similarly, individuals with sleep disordered breathing had an increased risk of developing all-cause dementia, AD, and vascular dementia.

### Smoking

Smoking may affect the risk of developing AD by various mechanisms. It is known that it is able to raise the generation of free radicals, increasing oxidative stress, and to promote pro-inflammatory action in the immune system, leading to the activation of phagocytes and consequently, additional oxidative damage. In addition, smoking may lead to cerebrovascular diseases, which increase the risk of AD [95, 96]. In a meta-analysis performed by Cataldo et al. (2010), an analysis of 8 case-control studies with affiliations with the tobacco industry suggested a protective effect of smoking in relation to AD (odds ratio (OR): 0.91, 95% CI 0.75–1, 10). In contrast, 14 cohort studies with no association with the tobacco industry demonstrated an increased relative risk for smokers (Relative Risk (RR): 1.45; 95% CI, 1.16–1.80) [97]. According to Durazzo et al. (2014), the sum of the evidence presented today in the literature is enough for the cessation of smoking to be recommended in order to reduce the incidence of dementia [96].

## Protective factors

### Cognitive reserve

It has been observed in many cases a discrepancy between the degree of brain damage found in histopathological analyses and the severity of cognitive decline. To explain these findings, the theory of cognitive reserve was proposed, which postulates that the gap between brain injury and clinical manifestations is attributable to cognitive reserve capacity. This can be subdivided into two models: brain reserve model or threshold, and cognitive reserve model and / or compensation. The first is based on the amount of available neural substrate (eg, brain size, synapses density or dendritic branching), while the latter focuses on the more efficient ability to use the preexisting brain network in healthy individuals and on the recruitment of more resources to support normal functioning in presence of brain damage [98].

Several elements are associated with a greater cognitive reserve, such as educational level, occupational activities, leisure activities, physical activities and the integrity of relationships network [98, 99]. A study conducted by Stern et al. (1994) [100] indicated that individuals with low level of schooling and low level of professional achievement had an approximately two-fold increased risk of developing dementia. Similarly, another study indicated that individuals with a higher level of leisure activities performance had a lower risk of developing dementia [101].

### Physical activity

A meta-analysis developed by Hamer & Chida (2009) [102] indicated that physical activity practice is able to reduce AD risk by 45%. This protective effect is related to several mechanisms, such as reduction of blood pressure, obesity and proinflammatory activity besides the improvement in lipid profile and endothelial function. In addition, adaptations that occur in response to exercise can lead to a better cerebral blood flow and, consequently, better oxygenation of important areas for cognitive function [102]. It is also believed that physical activity is able to prevent AD by increasing neurotrophic factors such as BDNF (Brain Derived Neurotrophic Factor), IGF-1 (Insulin-Like Growth Factor), VEGF (Vascular Endothelial Growth Factor), stimulating neurogenesis and synaptic plasticity; and by the reduction of free radicals in the hippocampus, as well as increase of superoxide dismutase and eNOS (endothelial nitric oxide synthase) [103]. Studies have shown that the practice of physical activities is capable of promoting an increase in hippocampal volume, in addition to increasing plasma BDNF concentrations in healthy elderly, indicating a possible neuroprotective effect. It was also reported that in the AD elderly, practice of physical activities correlates positively with the levels of BDNF [104], which is a growth factor associated with the development and survival of neurons and synapses [105].

### Diet

The relationship between the effects of diet and the risk of developing AD was based on certain patterns that were associated with lower or higher risk of developing AD [106]. As an example, Mediterranean diet is rich in unsaturated fats and antioxidants which confers a protection factor, as diets rich in saturated and trans fats and low levels of anti-oxidants are associated with higher risk of developing AD [106, 107]. Some dietary components are essential for neurocognition protection such as dietary fatty acids, including fish oil; antioxidants, such as vitamins E and C; fruits and vegetables; vitamins B6, B12 (cobalamine) and folate, in addition to caloric restriction [108]. Antioxidants are able to prevent damage caused by reactive oxygen species in addition to stabilizing the membranes; docosahexaenoic acid (DHA) helps clear the Aβ peptide and, together with choline and uridine, aid in the synthesis of the neuronal membrane [106]. Phospholipid composition is essential in neuronal membrane function. Thus, adequate intake of DHA, eicosapentaenoic acid (EPA), uridine monophosphate, choline, folate, vitamins B6, B12, C, and E, and selenium contributes to a better synthesis of phospholipids and, consequently, to synaptic function preservation and against neurodegeneration [106]. Nerve synapses consist mainly of neuronal membranes, and neuronal and synaptic losses observed in AD have been related to degeneration and alteration in the composition of these membranes [109]. Brain aging associated with changes in lipid composition is well studied for treatment and prevention purposes with phospholipids such as phosphatidylcholine and phosphatidylserine that could favor cognitive improvement [110]. The OmegAD study (a set of double-blind, placebo-controlled clinical trials involving AD patients which evaluated the effects of omega-3 fatty acids (n − 3 FAs) daily supplementation in patients with mild to moderate AD) showed that after six months, DHA (1.7 g) and EPA (0.6 g) supplementation demonstrated benefits such as preservation of cognitive performance, increase in plasma and CSF (Cerebrospinal fluid) levels of n^− 3^ FAs, DHA and EPA (and negative correlation between DHA and total / phosphorylated tau levels in CSF), reduction in cytokine release pro-inflammatory by blood peripheral mononuclear cells (PBMC), modulation in the expression of genes involved in the regulation of inflammation in PBMC, elevation in transthyretin plasma levels (a protein that binds to AB and which may influence its deposition in the brain), and increase in body weight and BMI. However, the literature data do not support the benefits of 3-FA supplementation in preventing cognitive decline in elderly subjects [111].

### Vitamin D

Epidemiological studies have observed a relationship between serum levels of vitamin D reduction, especially 25-hydroxyvitamin D, and AD development [112–114]. Vitamin D is an important steroid hormone that acts on calcium metabolism and bone regulation, and has some functions in central nervous system, such as regulation of neurotrophic factors, calcium homeostasis, acts on oxidative stress mechanisms, immune system modulation and inflammation [115]. In the case of inflammation, vitamin D deficiency causes an increase in the amyloidogenic pathway due to elevation of BACE1 and *APP* cleavage and decrease of Aβ degradation [116]. Briones & Darwish (2012) [117] reported a BACE1 and Aβ peptide reduction after vitamin D supplementation in elderly rats. It has also been observed that vitamin D acts on macrophages in order to promote clearence of Aβ peptide [118, 119]. In AD patients mutations were also observed in vitamin D receptor (VDR) gene, which would favor the onset of the disease [120]. To date, no large randomized clinical trial has been conducted on the effect of vitamin D supplementation on the cognition of AD patients. However, in smaller or cohort studies, the results of using high doses of vitamin D and cognitive improvement are divergent [121–124]. Vitamin D deficiency should be screened and supplemented in the elderly population due to its high prevalence, but this treatment is not specific for cognitive improvement.

### Estrogen (hormone replacement therapy)

Estrogen roles in sex organs are well understood, but it has recently been observed that local production of estrogen plays specific roles in tissues in which it is produced, with or without dependence on circulating estrogen [125]. Estrogen, especially estradiol, is able to prevent mitochondrial dysfunction in nerve cells, neuroinflammation and assist in DNA repair mechanisms [126], thus presenting neuroprotective effect [126, 127]. The results observed in epidemiological studies are inconsistent [128, 129]. Some studies have not observed a beneficial effect of hormone replacement therapy, estrogen or combination therapy on the risk of developing AD [130, 131]. Other studies reported a beneficial effect on cognition protection in women receiving hormone replacement therapy at different ages after the onset of menopause [132–135]. Inconsistent epidemiological findings, in addition to other factors such as increased risk of deep venous thrombosis, hormone replacement therapy is not recommended in order to prevent cognitive decline and AD development [136].

## Other relevant factors and conclusion

The main pathophysiological mechanisms of AD are amyloidosis and tau-related neurodegeneration, and have specific topographical and chronological pathways. For instance, brain amyloidosis starts in neocortical regions and then affects subcortical structures [137]. On the other hand, neurodegeneration first appear on locus coeruleus and then spreads through transentorrinal area and neocortical regions [137]. Cognitive and behavioral features of AD are significantly correlated to the topographical distribution of neurofibrillary tangles.

There is great variability in topographical patterns of pathological findings in AD, causing great phenotypical variability [7], with atypical presentations of the disease [138]. It is not clear how risk and beneficial factors may modulate the topographical progression of amyloidosis and neurodegeneration.

The effects of modifiable risk factors on cross-sectional cognition have been the target of multiple WRAP (The Wisconsin Registry for Alzheimer’s Prevention) investigations. This study has investigated risk factors for AD in middle age, since this phase of life is less studied in relation to the later stages of aging. However, this is a critical time because it is when the Alzheimer’s pathology begins and thus, when its trajectory can be modified through pharmacological approaches and / or lifestyle changes. Within this context, the WRAP study, reported by Johnson et al. (2018), suggest that social engagement, physical and cognitive activities, glucose regulation, stress and sleep, in addition to cardiovascular and metabolic risks are interventional parameters that may improve brain health and reduce the likelihood and severity of AD pathology. These authors conclude that a good health and a salutary lifestyle are factors associated not only with better cognition and brain structure but also the lower AD pathophysiologic burden [139].

The studies of genetic risk factors are important to better elucidate the pathophysiological processes in the development of AD. However, such factors are not passible to any intervention until now. Faced to this scenario, modifiable risk factors such as diabetes, hypertension and dyslipidemia and others previously mentioned should be closely monitored to prevent complications favoring cognitive decline or even to improve the quality of life of patients with AD. In this context, it should also be emphasized that factors considered protective, such as physical exercise, diet and cognitive stimuli should be strongly and widely encouraged, so that such theoretically preventive measures can be adopted by the population contributing to reduce risk of this disease. Since no current drug intervention can modify the pathophysiological mechanisms related to the development of this devastating disease, adoption of these measures constitutes an important strategy for clinical management in order to prevent or postpone cognitive decline.

## Abbreviations

AD: Alzheimer’s disease
AGEs: Advanced glycation end products
ApoE: Apolipoprotein E (ApoE)
APP: Amyloid precursor protein
Aβ: β-amyloid peptide
BDNF: Brain Derived Neurotrophic Factor
BMI: Body Mass Index
CSF: Cerebrospinal fluid
DHA: Docosahexaenoic acid
eNOS: Endothelial nitric oxide synthase
EPA: Eicosapentaenoic acid
HPA: Hypothalamic, pituitary and adrenal axis
HR: Hazard Ratio
IGF-1: Insulin-Like Growth Factor
n − 3 FAs: Omega-3 fatty acids
NFT: Neurofibrillary tangles
PBMC: Peripheral blood mononuclear cells
P-tau: Phosphorylated tau protein
VDR: Vitamin D receptor
VEGF: Vascular Endothelial Growth Factor
